# 

**Published:** 1888-07-14

**Authors:** 


OUR grandfathers used to cure the headache by kissing a pretty girl. Some of these old-time remedies are difficult to improve on.

**Special Notice to Newsagents, Advertisers, and the Trade.**

**CHANGE OF OFFICE AND PUBLISHER.**

Notice is hereby given that the Office of The Hospital will henceforth be at 38, Old Jewry (Cheapside end), E.C., to which address all communications should be sent. Mr. A. Doig has been appointed Advertising Agent, and Mr. J. H. S. Hanning, Manager. All Cheques and Post Office Orders should be made payable to J. H. S. Hanning, 38, Old Jewry, E.C., crossed Lloyds, Barnetts, and Bosanquets Bank (Limited).

**To Secretaries and Matrons.**

The Editor will be glad to have a copy of the Regulations, and also of every Report issued from the Press of Asylums, Hospitals, Convalescent and Nursing Institutions, as well as those of other Charities, so soon as they are published, and an early copy in duplicate of all appeals for funds. Notices of Annual and other Meetings, Festival Dinners, etc., will be welcomed. All such communications should be addressed to *The Editor, The Lodge, Porchester Square, London, W.* Any superintendent, secretary, matron, or other chief official who will regularly send such documents and information will be registered to receive free of cost a cover for binding The Hospital in half-yearly volumes on sending name and address to the publisher.

Alcohol versus Race.—While alcohol does not seem to have produced any racial deterioration in white races, or rather in the Indo-Germanic family, it acts differently upon Asiatice and dark races. Alcohol in any quantity seems to set most Asiatics—the Jews are an exception—on fire, to produce an irresistable craving for more, and to compel them to go on drinking until they are sunk in a stupor of intoxication. They appear to delight but little in the exhilaration produced by partial inebriety, and to seek always a total release from consciousness and its oppressions. The condition of "dead drunkenness," which few even of drinking northerners enjoy, is to them delightful. "I not drinkee for drinkee," said the Madras man; "I drinkee for drunkee." Alcohol is therefore to such races an intolerable evil, and its consumption by them is in the eyes of all strict moralists an immorality.

### Amusements with Profit for all Ages.

**Rules.**—All answers must be addressed to the Prize Editor, The Hospital, 38, Old Jewry, Cheapside, London, E.C., not later than the *first* post on Thursday next. The full name and address must in all cases be given, but a *nom de plume* may be added for publication. Only one side o the paper must be written on.

### FOR MEN AND WOMEN.

### First Quarterly Poetical Competition.

Commenced May 26th, 1888 ; ends August 25th, 1888.

This quarter a PRIZE of ONE GUINEA will be given to the competitor who gains the highest number of marks for original poems, on any subjects suitable for insertion in this paper. All contributions sent in will be credited according to merit, eighteen marks being the highest score for any one contribution. This competition to alternate weekly with the set acrostic competition.

### Acrostic Competition.

This quarter the original acrostic competition will be discontinued, and A PRIZE of HALF-A-GUINEA will be given to the competitor who gains the highest score for solution of acrostics set. *One mark* only will be given to the competitors who solve all the lights of each acrostic.

Exception will be made in the case of *quotation acrostics*, when *two marks* will be given, *one* for the correct solution of all *lights*, and *one* mark for the *source* of the quotations.

This week credit will be given for

### No. 4.—Original Poem.

### Results of No. 3.—Original Poem.

Dodo (15), K. M. (15), Patience (14), Hesperornis (12).

### Original Poem.

Within the garden of each human heart,
Where trodden past with trackless present blends,
There ought to be a little sacred part,
The weal whereof on secret care depends—
A pleasant plot, where memory renews
The hours of kindly thought and gentle deed
Given to others with untiring ruth,
Not of quick impulse, but well-nurtured seed,
Leading us backward to a hopeful youth,
Through the long vista of the sober years.

But if a consciousness of love of change,
Of noble aims laid by, should haunt your breast,
Of fitful searchings for the new and strange
Should mar your musings and disturb your rest ;
Still, do not turn away your weary thought,
Leaving old weeds to tangle thicker still,
But think awhile upon your garden plot,
And use the present wisely. Out of ill
And weary suffering may some good be got.
You may return to life with freshened will,
And leave behind old failures and old fears.

A. M. E.

### THE WORD COMPETITION.

Third Quarterly Competition commenced

May 5th, 1888 ; ends August 4th, 1888.

Three prizes of one guinea, 15*s.* and 10*s.* 6*d.* will be given each quarter to the three competitors who obtain the highest number of marks by making the largest number of words derived from the word or phrase set for anatomical dissection ; that for this, the *tenth* week of the quarter, being

"Pension."

Observe.—Proper names, abbreviations, foreign words, words of less than four letters, and repetitions are barred ; plurals, and past and present participles of verbs are allowed. Nuttall's *Standard* dictionary only to be used.

N.B.—All words sent in must be arranged *alphabetically*, with correct total affixed.

### Results of Eighth Week.

*Moonshine.*—Reldas (46), Time (46) Patience (46), Lightowlers (46), Lauriston (46), Sym (45), Dodo (43), Dollie (40), Oliver (40), Puff (36.)

### THE CHILDREN'S CORNER.

PRIZES.

FOR MOST CORRECT ANSWERS TO PUZZLES.

This Commenced October 1st, 1887.

One Pound a Month.

A PRIZE OF THREE GUINEAS

to the Competitor who shall have sent in the largest number of correct or best answers during the twelve months.

### Puzzles.—Second Week.

No. 5. I am a word of five letters. My first, minus my fifth, will leave my second ; my fifth divided by my first will produce my fourth ; and five times my first added to five times my fifth will make my third ; my whole is funny.

No. 6—Diamond Puzzle. 1. A. Consonant. 2. A British beverage. 3. A twist of hair. 4. A town in Scotland. 5. A modern physician, 6. A quadruped. 7. A consonant.

a a a a c e e g g g g i l l l o o p p s t t w w w

No. 7. Geographical Charade.—My *first* is never old ; my *second* faces the north ; my *third*, though a part of Great Britain, is neither in England nor Scotland ; my whole is a beautiful and productive country.

No. 8. Geographical Acrostic.—1. Seaport town in Durham. 2. A town of Japan. 3. A seaport of France with famous harbour. 4. A river and town in Ayrshire. 5. The celebrated treaty of peace in 1802. Initials give the name of a district in Asiatic Turkey, and finals something which comes from it.

### Results of Fourth Week Puzzles.

No. 15. Arithmetical Puzzle.—He was 60 years old.

$$42 \times 42 = 1764$$

$$42 + 18 = 60$$

No. 16. Double Acrostic.

| | | |
|---|---|---|
| W | orkso | P |
| O | rinoc | O |
| R | ocheste | R |
| C | arnati | C |
| E | astbourn | E |
| S | caw Fel | L |
| T | arragon | A |
| E | —— | I |
| R | oue | N |

No. 17. Square Word.

| | | | |
|---|---|---|---|
| L | A | M | E |
| A | C | I | D |
| M | I | N | E |
| E | D | E | N |

No. 18. Charade.—Whale-bone = Whalebone,

*All right*—A. C. K., Gem, Kensington. *Three right*—Hercules, Scrooge, R. H. *Two right.*—Alsie, Poodle, Thanet. *One right*—Plummy Fluff.

### Results of Ninth Monthly Competition.

This month we award the prize of £1 to Kensington. A.C.K. and Scrooge have both gained the same total of marks, but they are debarred from participating in the prize through having already received one prize since October last. We congratulate them upon the perseverance and regularity with which their weekly answers have been sent in during the past nine months,

The prize will be forwarded to "Kensington" (Master Paul Methven, The Acorns, Oakleigh Park, N., aged 10½).

### Conditions.

I. Every paper sent in must be clearly written, and one side only must be used. All competitors must mark at the head of their papers the number of their competition.

II. Each paper must be signed by the author with his or her real name and address. A *nom de plume* may be added, if the writer does not desire to be referred to by us by his real name. In the case of all prizewinners, however, the real name and address will be published.

III. All communications relating to prize competitions should be addressed to "The Prize Editor, The Hospital, 38, Old Jewry, London, E.C." Competitors are requested not to include in letters, etc., to the Prize Editor communications on matters of other business. All letters must be fully prepaid.

IV. The Prize Editor of The Hospital is the sole judge of the competitions, and his decision is in all cases final and without appeal.

V. The Prize Editor reserves the right to publish any paper sent in, whether it receives a prize or not. He does not hold himself responsible for any MSS. sent, nor can he undertake to return rejected competitions.

VI.—All children resident at school are requested to forward their *home* address in addition to the school one, when entering for the "Children's Corner" Competitions.

**Notice to all Correspondents.**—N.B.—All letters referring to this page, which do not arrive at 38, Old Jewry, Cheapside, London, E.C., by the *first post on Thursdays*, and are not addressed PRIZE EDITOR, will in future be disqualified and disregarded.

No one will be permitted to win the Monthly Prizes twice in any one year, the Annual Prize being offered especially to prevent this.

